# A Retrospective Study Comparing Crossed and Lateral Wire Configurations in Paediatric Supracondylar Fractures

**DOI:** 10.2174/1874325001711010432

**Published:** 2017-05-30

**Authors:** Murtaza K. Khwaja, Wasim S. Khan, Pinak Ray, Derek H. Park

**Affiliations:** Department of Trauma and Orthopaedics, Royal Free Hospitals NHS Trust, Barnet Hospital, Barnet, London, UK

**Keywords:** Retrospective, K-wire, Configuration, Supracondylar fractures, Iatrogenic nerve injury

## Abstract

**Background:**

Supracondylar fractures are common in children and are associated with significant morbidity.

The purpose of our study was to assess and compare the clinical and radiological outcome of management of supracondylar fractures by both wire configurations, along with identifying factors that predispose to complications.

**Materials & Methods:**

We retrospectively reviewed all paediatric cases admitted with a supracondylar fracture over a five year period. We reviewed case notes, theatre records and radiographs to determine the age of the patient, classification of fracture, treatment method, delay to theatre, duration of surgery, wire configuration, Baumann´s angle, radiocapitellar alignment, anterior humeral alignment and complications.

**Results:**

During the five year period we admitted 132 patients and complete notes were available for 123 patients for analyses. For all the patients managed with wire stabilisation 23% developed complications, including 13% with significant complications including nerve injuries and fracture displacements. All five nerve injuries had crossed wires, whereas all for fracture displacements had lateral wires. Baumann´s angle was 76.7 degrees in the group with no complication and 72.2 degrees in the significant complication group (p=0.02). Radiocapitellar line and anterior humeral line were not satisfactory in 5% and 15% of the group with no complications, and 17% and 33% of the group with significant complications.

**Conclusion:**

We found more complications in lateral pinning configurations, although all nerve injuries were in patients with crossed wire configurations. The factors we believe are associated with a higher likelihood of complications are inadequate post-operative radiological appearance.

## 1 INTRODUCTION

Supracondylar humeral fractures are common in the paediatric population and account for almost 70% of elbow fractures [1-3]. The incidence peaks between the ages of 5 - 8 years [4, 5]. These fractures are either extension or flexion type with varied mechanism of injury; extension type fractures account for 96-99% of all supracondylar fractures [6, 7].

Supracondylar fractures are commonly classified based on the Gartland system of classification, where they are divided into three types; Type I being non-displaced, Type II being displaced but with an intact posterior cortex and Type III being displaced and without any cortical contact [4], although there are more recent modifications [8, 9]. Type I are generally treated nonoperatively in an above-elbow plaster cast with the elbow in 60-90 degrees flexion for three weeks with radiographs to check for displacements [10]. Type II and Type III are generally managed with closed reduction and pinning in order to prevent malunion [10]. Displaced supracondylar humeral fractures can present with vascular [11, 12] and/ or neurological [3, 13, 14] compromise in up to a fifth of cases.

**Fig. (1):**
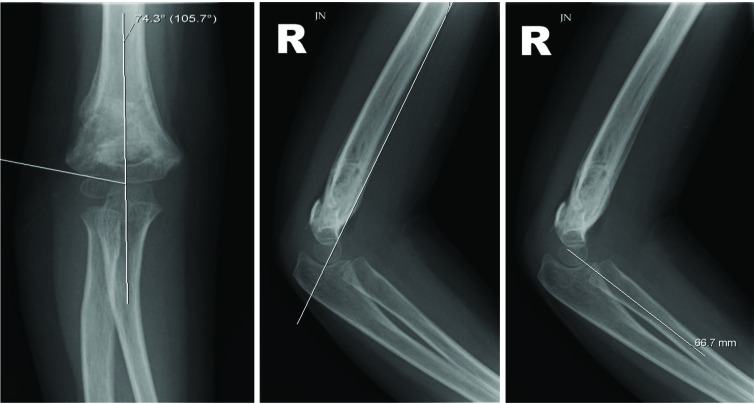
The radiographs show the calculation of Baumann’s angle (**a**), and the assessment of the anterior humeral line (**b**) and the radiocapitellar line (**c**).

Along with a posterior fat pad sign in Type I fractures, three radiographic parameters used to evaluate a supracondylar fracture are the Baumann’s angle, anterior humeral line and radiocapitellar line [15]. The Baumann’s angle is around 75 degrees [16], and the anterior humeral and radiocapitellar lines should cross the capitellum through its middle third on a lateral x-ray [15] (Fig. **1**).

**Fig. (2):**
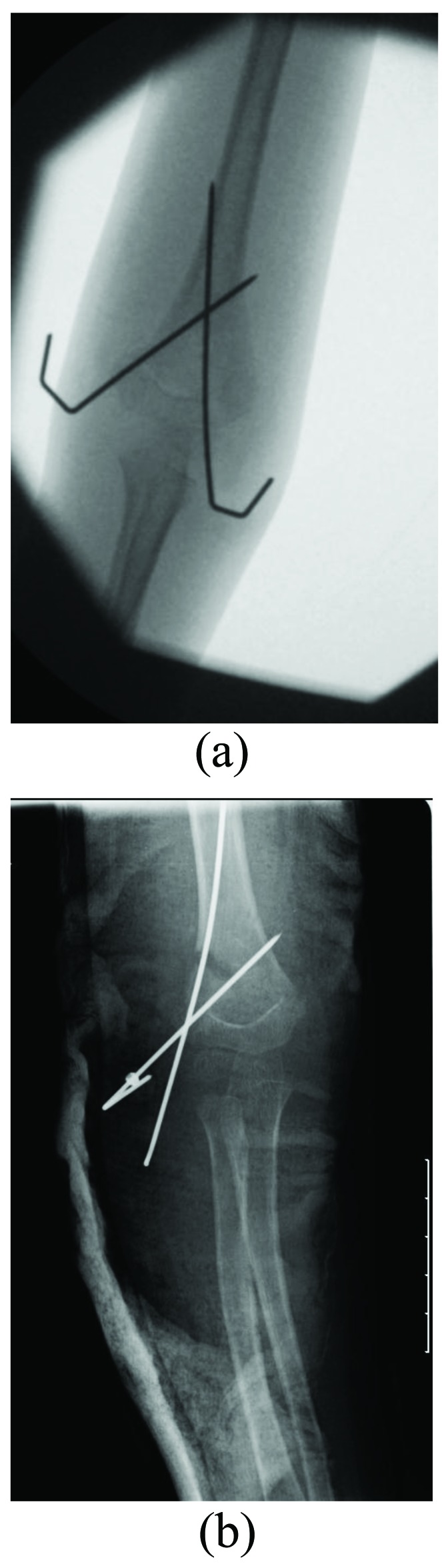
Radiographs showing the crossed (**a**) and lateral (**b**) K-wire configuration.

Percutaneous K-wiring is the most widely advocated method to stabilise displaced supracondylar fractures after reduction. There is no clear consensus on the configuration of K-wiring. Commonly used configurations include a crossed configuration with a medial and a lateral K-wire, and lateral configuration with two lateral K-wires [17] (Fig. **2**). Both have advantages and disadvantages. The crossed wire configuration is biomechanically more stable, especially when resisting axial forces [18-20]. Brauer *et al.* [17] conducted a systematic review showing a 58% lower risk of residual deformity or loss of reduction with the crossed wire configuration compared with the lateral configuration. The ulnar nerve however is at risk of iatrogenic injury from the medial K-wire [18, 21]. Brauer *et al.* [17] also reported a five-fold risk of iatrogenic ulnar nerve injury with the crossed configuration as opposed to the lateral configuration. A further systematic review of randomized controlled trials comparing the two wire configurations in extension type Gartland type III fractures however found no difference in clinical or radiological outcomes [22].

The purpose of our study was to assess and compare the clinical and radiological outcome of management of supracondylar fractures by both wire configurations, along with identifying factors that predispose to complications.

## 2 MATERIALS AND METHODS

We retrospectively reviewed all supracondylar fractures in children between the ages of 2-15 years old that were admitted to our unit between November 2009 and November 2014. We carried out a review of case notes and theatre records to determine the age of the patient, time to operation theatre and duration of surgery. We carried out a review of patients’ radiographs to determine the type of Gartland fracture, Baumann’s angle, radiocapitellar alignment and anterior humeral line post-intervention/surgery. Radiographs were also used to determine the type of wire configuration used. We performed students t-test for statistical analyses and a p value of <0.05 was considered significant.

## 3 RESULTS

There were 132 patients with Gartland type II and type II fractures admitted to out unit over the five year period and complete notes were available for 123 patients. Out of 123 patients, 12 were managed nonoperatively, and 13 were managed with a manipulation under anaesthesia. None of these patients had any complications. All the remaining 98 patients were treated with K-wiring, either crossed or lateral. They had a mean age of 6.1 years (SD 2.6 years). These included 61 type II and 37 type III fractures. Fifty-nine patients were managed with crossed K-wires and 39 were managed with lateral K-wires. The ages and fracture types were not significantly different between the two wire configuration groups.

Out of these patients managed with wire stabilisation 23% (22 patients) developed complications, including 13% (13 patients) with significant complications including nerve injuries (five patients) and fracture displacements (four patients). Out of the five nerve palsies, two were ulnar nerve palsies, two were radial nerve palsies, and one was a median nerve palsy. The mean age, classification, time to theatre and duration of surgery were not significantly different between the patients with and without complications (p > 0.05). The rate of complications was not different between the two groups; 33% in lateral wire configuration compared with 26% in those treated with crossed wires. Five of the significant complication patients had lateral wire configuration whereas the other eight had crossed wires. All five nerve injuries had crossed wire configuration, whereas all four fracture displacements had lateral wire confirmation. The mean Baumann’s angle was 76.7 degrees in the group with no complication and 72.2 degrees in the significant complication group (p=0.02). The radiocapitellar line and anterior humeral line were not satisfactory in 5% and 15% of the group with no complications, and 17% and 33% of the group with significant complications.

## 4 DISCUSSION

A systematic review [22] in 2012 looked at randomized controlled trials comparing efficacy of crossed versus lateral K-wire fixation in extension type Gartland type III fractures and identified four studies but none was level 1. In the first randomised controlled trial by Tripuraneni *et al.* [23] patients were randomized preoperatively but the final decision was up to the operating surgeon based on intraoperative findings. There was no blinding, and there were only 20 patients in each group with seven patients lost to follow-up. The authors performed intention-to-treat analyses and found no statistically significant difference in complication rates, range of motion, or radiographic alignment (Baumann’s angle and humerocapitellar angle). The authors took steps to avoid iatrogenic nerve injuries. The ulnar nerve was palpated intraoperatively and if it easily subluxed anteriorly, medial pin placement was abandoned. In addition, medial K-wires were tested with a nerve stimulator as described by Wind *et al.* [24]. The authors reported one ulnar nerve injury in a patient that did not have intraoperative nerve excitability. The injury resolved after seven months. The second study was by Foead *et al.* [25] looking at 55 patients, but it lacked postoperative baseline radiological assessments and all reductions were assumed to be anatomical, limiting follow-up assessments. They reported an overall ulnar nerve iatrogenic injury rate of 12.72% consisting of five crossed configuration patients and two lateral configuration patients. This difference was not statistically significant. The authors also noted a radial nerve palsy in the lateral wire group postoperatively. The authors found no statistically significant difference in the alignment, range of movement or Baumann’s angle between the two groups.

Two further randomised controlled trials were performed by Gaston *et al.* [26] and Kocher *et al.* [27] looking at 104 and 52 patients, respectively. Although both extended the elbow to over 90 degrees prior to inserting the medial K-wire to avoid injury to the ulnar nerve, only Kocher *et al.* made an incision over the medial epicondyle. Gatson *et al.*. treated four crossovers as intention-to-treat whereas Kocher *et al.* excluded crossovers. Both studies looked at ulnar nerve injuries and changes in Baumann’s angle and humerocapitellar angle. Only Gatson *et al.* looked at range of movement and loss of carrying angle. Neither study found a significant difference in the clinical or radiological parameters between the two wire configurations. Although the definition for loss of reduction varied between the two studies, neither identified a difference between the wire configurations in relation to loss of reduction. Although Kocher *et al.* did not report any nerve injuries, Gaston *et al.* reported two cases with the crossed configuration. They report one case of ‘tenting of the nerve’ with incomplete recovery at three months follow-up, and one case of ‘pin indenting the nerve’ at 90 degrees of elbow flexion with complete recovery at three months.

Our results suggest that both wire configuration patters are valid but the complication profile varies. In our study fracture displacement was seen only with lateral wiring, and nerve injuries only seen with crossed wires. Our results support those of biomechanical studies [19, 21, 28] and clinical reviews [17] showing the crossed configuration is a more stable construct. Our results also support a previous meta-analysis [29] looking at outcomes following lateral wire configuration showing a significantly higher risk of iatrogenic ulnar nerve injury compared with crossed wire configuration. No previous randomised controlled trial has however identified a significant difference in clinical or radiological outcome between the two wire configurations. Another important finding in our study was the statistically significant difference in the Baumann’s angle in the group with no complication and the complication group. We also showed that the radiocapitellar line and anterior humeral line were not satisfactory in a higher proportion of patients in the group with significant complications. These findings highlight the need to obtain adequate reduction to reduce the chances of complications.

Our study has limitations. It is a retrospective study where the procedure as carried out by a number of different surgeons. We did not look at the three lateral wire configuration or K-wire sizes. There is some evidence that three lateral wires produce a more stable construct than two lateral wires [26, 28], and that 1.6mm wires provide more stability than 1.25mm wires in all configurations [30, 31]. Our findings nevertheless are supported by the broader literature and by biomechanical studies. Although there are four randomised controlled trials comparing the outcome of crossed versus lateral K-wire configuration in supracondylar fracture that found no significant difference, they all have limitations [23].

## CONCLUSION

We found more complications in lateral pinning configurations, although all nerve injuries were in patients with crossed wire configurations. The factors we believe are associated with a higher likelihood of complications are inadequate post-operative radiological appearance. We suggest that future randomised controlled trials are sufficiently powered with larger patient numbers to detect significant differences in clinical and radiological outcomes.
